# Rates and timeliness of treatment initiation among drug-resistant tuberculosis patients in Nigeria- A retrospective cohort study

**DOI:** 10.1371/journal.pone.0215542

**Published:** 2019-04-25

**Authors:** Charity Oga-Omenka, Christina Zarowsky, Aderonke Agbaje, Joseph Kuye, Dick Menzies

**Affiliations:** 1 School of Public Health of the University of Montreal (ESPUM), Montreal, Canada; 2 Public Health Research Institute of the University of Montreal (IRSPUM), Montreal, Canada; 3 Institute of Human Virology, Abuja, Nigeria; 4 National TB and Leprosy Control Program, Federal Ministry of Health, Abuja, Nigeria; 5 McGill University International TB Centre, Montreal, Quebec, Canada; 6 Department of Epidemiology and Biostatistics, McGill University, Montreal, Canada; Department of Internal Medicine, Federal Teaching Hospital Abakaliki, Ebonyi State, Nigeria, NIGERIA

## Abstract

**Background:**

There were an estimated 580,000 new cases of multidrug/rifampicin resistant TB (DR-TB) in 2015, and only 20% were initiated on treatment. This study explored health system and patient factors associated with initiation and timeliness of treatment among DR-TB patients in Nigeria, ranked 4^th^ globally for estimated TB cases in 2015.

**Methods:**

A retrospective cohort study using 2015 diagnosis and treatment data from the Nigerian TB program electronic records examined “treatment ever received” (yes/no) and “treatment within 30 days” (yes/no). We compared health system and patient characteristics using binomial logistic regression, while controlling for confounders.

**Results:**

Of 996 patients diagnosed nationwide in 2015 (aged 0–87 years, median 34), 47.8% were never treated. Of those treated (n = 520), 51.2% were treated within the 30 days prescribed in the National treatment guideline. Healthcare facility locations were significantly associated with ever receiving treatment and timely treatment. Predictors of timely treatment at the national level also included level of care and patient treatment history. The South-West zone, where DR-TB programs started, showed overall better access to DR-TB healthcare.

**Conclusions:**

Healthcare facility geographic locations were significantly associated with treatment initiation and timeliness. Significant regional differences in access to DR-TB care in Nigeria persist, reflecting uneven contexts for national DR-TB treatment rollout.

## Introduction

There were an estimated 10.4 million incident TB cases worldwide in 2015 (25% in Africa) and 580,000 DR-TB new cases.[1] Although DR-TB survival rates are lower and treatment more difficult, studies have suggested that treatment outcomes are optimized by timely diagnosis and treatment, as mortality rate is highest within the first month of diagnosis.[2–4] The World Health Organization (WHO) recommends treatment initiation within 4 weeks of diagnosis.[5]

In 2015, only 20% of new DR-TB cases were initiated n treatment.[1] Five countries, including Nigeria, made up more than 60% of this gap in treatment.[1] Nigeria, ranked 4th globally for estimated TB cases (586,000 incident cases and 29,000 new DR-TB cases), is one of 14 countries categorized by the WHO as having overlapping high burdens of TB, DR-TB and HIV.[1,6] Of these, however, only 90,584 TB and 1,241 drug resistant incident cases were notified in 2015, giving a case detection rate (CDR) of 15% and 4.2% respectively, some of the lowest globally.[1] This highlights major difficulties in accessing TB diagnosis and treatment.

Nigeria commenced free DR-TB treatment in 2010 with a hospital-based care model and adopted the use of Xpert MTB/RIF technology in 2011, leading to an increase in case detection.[7–8] Community DR-TB treatment initiation commenced in 2013 and was scaled-up in 2015.[9] Available literature does not clearly document how long DR-TB patients are waiting to be started on treatment, nor the socio-demographic differences between diagnosed and untreated patients and those who are treated.

We conducted a retrospective review of national diagnosis and treatment electronic medical records (EMR) between January to December 2015 in order to estimate the proportion and characteristics of patients not started on treatment within one month after diagnosis.

## Materials and methods

### Study setting

**Fig 1:**
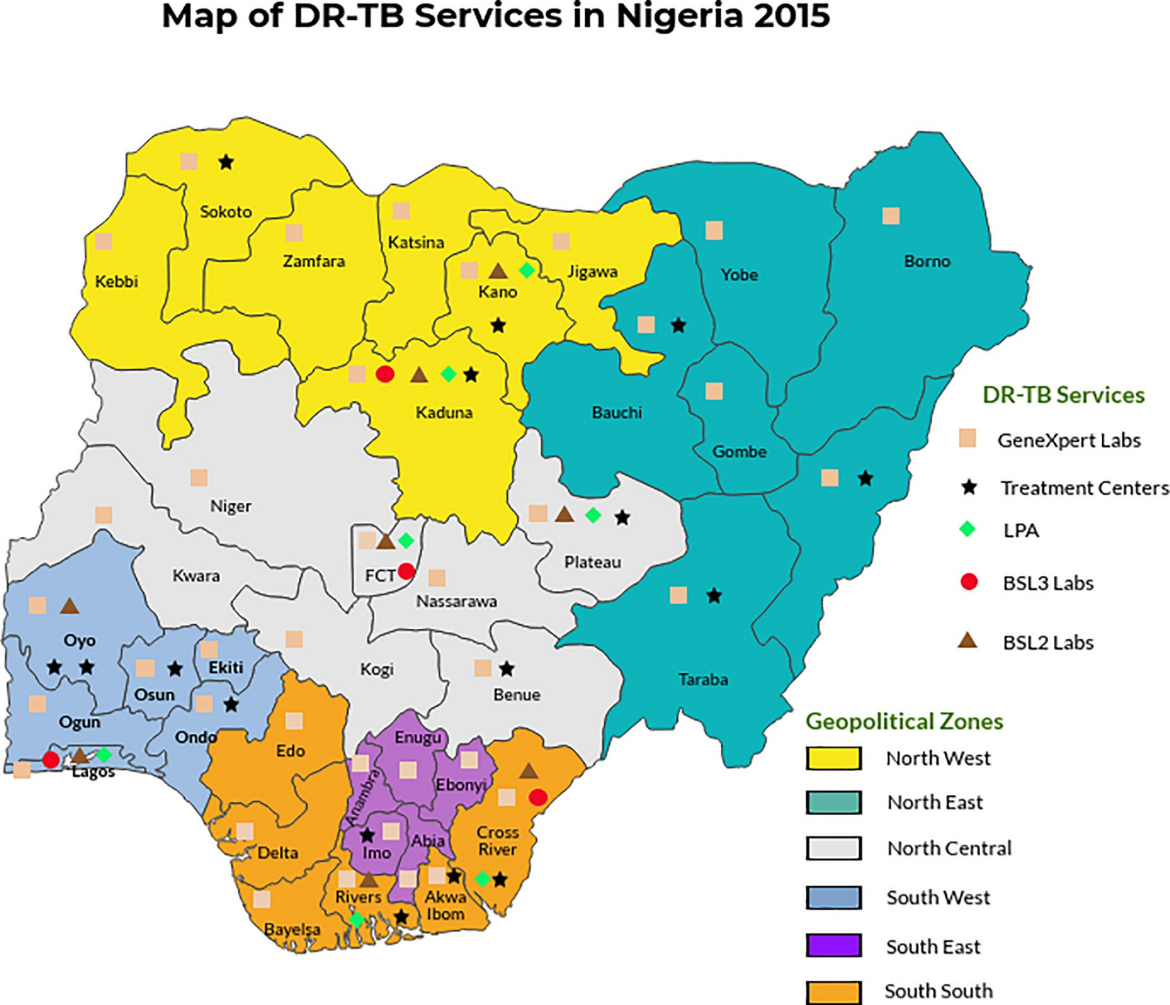
Map of Nigeria showing coverage of DR-TB Services as at November 2015.

Nigeria, the most populous country in Africa with an estimated population of 183 million people in 2015,[10] has 37 States divided into 6 geopolitical zones: North-Central, North-East, North-West, South-East, South-South and South-West (Fig 1). Since 2011, the Federal Ministry of Health has implemented DR-TB care, with support from the Global Fund and other partners in 28 tertiary and secondary facilities within 27 States.[11]

The national treatment guidelines recommend that patients be placed on treatment within one month of diagnosis.[2,3] A web-based system for GeneXpert reporting, the GxAlert, was introduced in 2012 which facilitates the flow of patients, samples, and results at facility level by transmitting instant SMS results to clinicians and patients.[12,13]

By the end of 2015, the National TB and leprosy control program (NTBLCP), reported 201 GeneXpert sites[14] and a total of 52,219 GeneXpert tests performed using 176 machines. Of these, 11,745 (22.5%) were positive for Mycobacterium tuberculosis, with 1,264 Rif-resistant.[12]

Similar to the DOTS strategy before it, treatment for DR-TB in Nigeria began in the SW zone in 2010 before being scaled to other regions. The SW region had the only national reference laboratory for several years; and at the end of 2015, had the highest numbers of gene expert machines installed, patients diagnosed, hospital beds and patients enrolled for DR-TB treatment.[9, 14–15] The National TB prevalence survey showed that the SW zone had the highest incident cases of TB,[11] and several studies have indicated that the zone also has the highest incidence of DR-TB.[15–17]

### Definitions

Time to treatment initiation (TTI) was defined as the interval between the dates of diagnosis of DR-TB and the initiation of anti-TB treatment. “Untreated” was defined as patients diagnosed in 2015 who had not started treatment by the end of August 2017. “Treated” patients were further categorised into “Early” if initiated between 0–30 days and “Late” if more than 30 days.

Two types of DR-TB facilities- diagnosis and treatment—were further categorized. Types of diagnosis facility included “Reference laboratories” (2 national and 6 zonal reference laboratories), “Federal hospital” (tertiary facilities), “State-owned” (secondary facilities), “Primary Hospital” (Primary health care administered by the local government areas (LGA)) and “Private Hospital”.[18] Levels of treatment facilities included “Facility-based” (for patients who started DR-TB treatment as in-patients) and “Community-based” (for those who started treatment as out-patients).

The geographical size classification was based on the 2015 estimated population of each LGA in Nigeria.[10, 19–20] LGAs with more than 400,000 were classified as “Urban”, between 250,000 and 400,000 as “Semi-Urban” and less than 250,000 as “Rural”.

Case detection rate, patient treatment history and type of resistance were classified based on the WHO and national DR-TB definitions.[21–23]

### Study design

A retrospective cohort was identified of diagnosed DR-TB patients in 2015 from the Nigerian National TB program EMR. Patients were tracked by checking treatment initiation records up to August 2017 (20–32 months after diagnosis).

The first data source for this study was the GxAlert data, which included patients diagnosed with Mycobacterium tuberculosis (MTB) and resistance to rifampicin (RIF) using GeneXpert MTB/RIF technology in 2015.

The second data source, the e-TB Manager is a comprehensive web-based record of all patients placed on TB treatment.

All valid entries for diagnosed patients who were diagnosed (GxAlert database) and treated (e-TB Manager Database) in 2015 were analyzed to estimate treatment waiting times and to identify socio-demographic differences between treated and untreated patients among the diagnosed populations.

### Data linkage process

**Fig 2:**
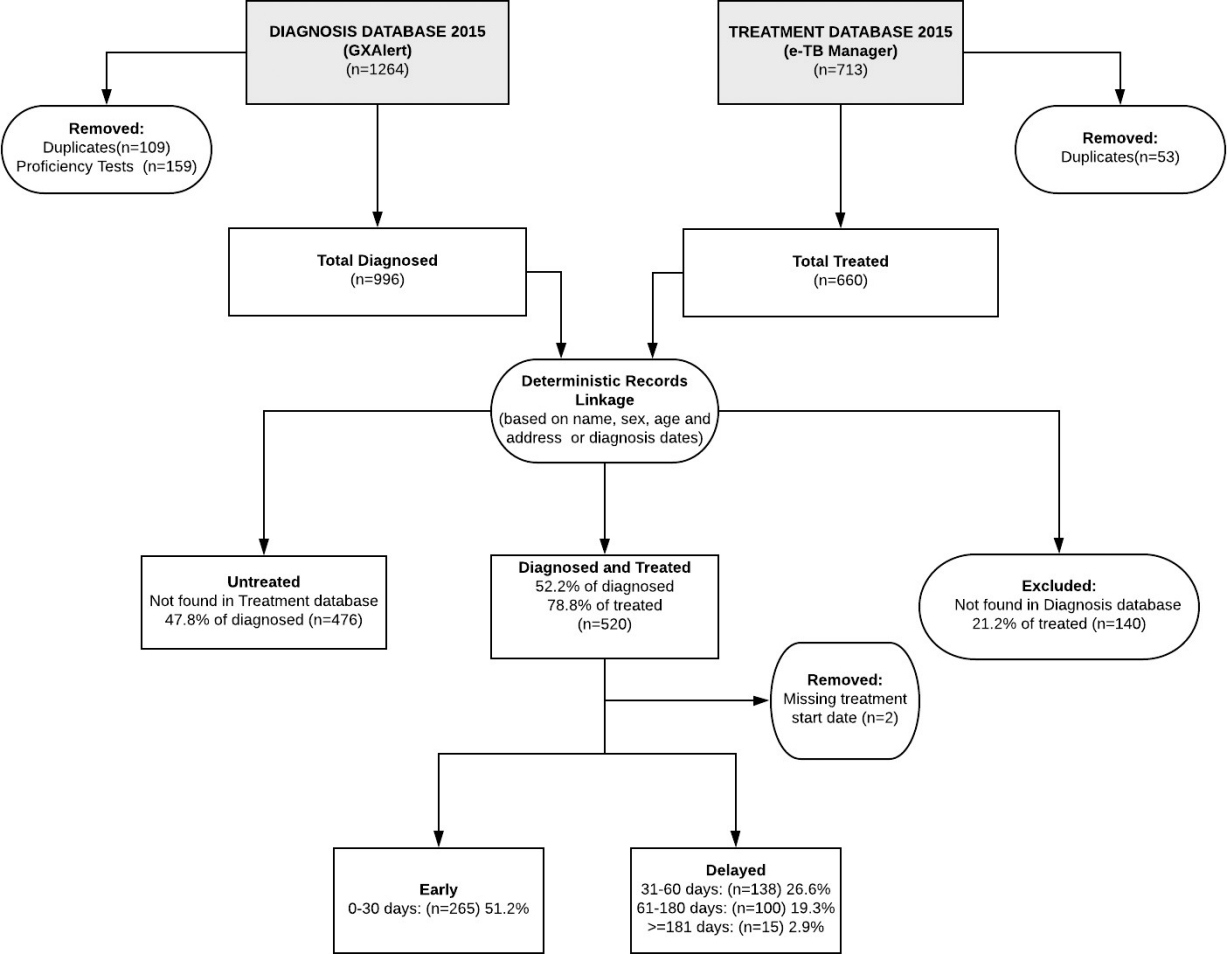
Flow diagram of study recruitment strategy.

We used a deterministic approach for records linkage based on surname, first names, sex, age and address from the different registers because they did not contain unique patient identifiers. As shown in Fig 2, among the 996 cases of TB diagnosed and registered in the Diagnosis (GXAlert) database in 2015, 52.2% (n = 520) were also registered in the treatment (e-TB Manager) database and were matched.

A proportion of the records in both databases could not be matched. For patients found in the diagnosis register and not in the treatment register (n = 476), they were classified as ‘untreated’ and compared with patients diagnosed and treated. Patients found in the treatment database and not in the diagnosis database were excluded (n = 140), with a description of their basic characteristics presented in Table 1.

**Table 1:** Description of basic characteristics of study and excluded population.

Characteristic		Study Population	Excluded*
TOTAL		*996* *n (%)*	*140* *n (%)*
Age			
	Children (0–19)	80 (8.0)	5 (3.6)
	Adults (20–59)	870 (87.3)	125 (89.3)
	Seniors (≥60)	43 (4.3)	10 (7.1)
	*Missing*	*3 (0*.*3)*	*0*
	Range	0–87	15–77
	Mean (SD)	35.45 (12.79)	38.37 (12.11)
	Median	34.00	37.00
Sex			
	Female	349 (35.0)	32 (22.9)
	Male	647 (65.0)	108 (77.1)
Geopolitical Region			
	South West	286 (28.7)	53 (37.9)
	North Central	250 (25.1)	18 (12.9)
	South South	143 (14.4)	21 (15.0)
	North West	116 (11.6)	36 (25.7)
	South East	107 (10.7)	9 (6.4)
	North East	94 (9.4)	3 (2.1)
Urban/Rural			
	Urban	526 (52.8)	83 (59.3)
	Semi-Urban	290 (29.1)	33 (23.6)
	Rural	180 (18.1)	21 (15.0)
	*Missing*	-	4 (2.1)
Type of Diagnosis Facility**			
	National/Zonal Laboratory	316 (31.7)	-
	Federal Hospital	213 (21.4)	-
	State-owned Hospital	397 (39.9)	-
	Primary Hospital	16 (1.6)	-
	Private Hospital	54 (5.4)	-

* These 140 patients were registered in the treatment database (e-TB manager) as diagnosed in 2015 but could not be traced in the diagnosis database (GxAlert).

** The treatment database, where the 140 patients were found, did not have information about the type of diagnosis facility

### Statistical analysis

Diagnosis and treatment data were analysed at the national and at the SW regional levels. Excel data files sourced from the GxAlert and E-TB manager were cleaned and validated before analysis. Data preparation involved matching sex to names (missingness reduced from 10.0% to 3.3%) and the use of multiple imputation to handle missing data, specifically for age (missingness reduced from 21.7% to 0.3%) and sex (from 3.3% to 0.0%), after a determination of missing completely at random (using the Little’s MCAR test). Missing values were imputed using the automatic settings of the multiple imputation method and random number generator (Mersenne Twister). A total of 10 data sets of imputed values were created and used in subsequent analyses for both databases, and pooled results reported. Standard descriptive statistics were used to characterize the cohort in both databases. We measured the variance inflation factor (VIF) in the variables in order to detect and exclude the existence of collinearity, and subsequently logistic regression analyses. Univariate binary logistic regressions were used to estimate crude odds ratios and their 95% confidence intervals (CI) for the association between socio-demographic differences (age, sex, type of facility, geopolitical regions) and two outcome variables—treatment initiation (untreated versus treated), and timely treatment (early versus late treatment) among those diagnosed in 2015. Multiple logistic regression models were used to identify predictors of treatment initiation and its timeliness. Cox proportional hazards regression was used to simultaneously assess the effect of several socio-demographic factors on TTI, where survival time was the TTI in days. Patients were censored when not placed on treatment as at August 2017 (≥601 days) Statistical significance for all analysis was pegged at 5% level (0.05). Violation of proportional hazard assumptions was checked by examining the Log−log plots with the aim of excluding any covariates which violated the assumptions. All statistical analysis was done using the SPSS software, version 24.

### Ethics statement

Ethical approval was obtained from the National Health Research Ethics Committee of Nigeria (NHREC/01/01/2007). Approval was also received from the Research Ethics Committee (CER) of the University of Montreal Hospital (17.060).

## Results

### Characteristics of DR-TB patients

From January to December 2015, a total of 996 entries out of 1264 unique diagnosis entries were selected for analysis, after duplicates and routine proficiency tests were excluded. Diagnosed patients’ records were matched with treatment records using patient name, sex, age and address, as well as the test and treatment dates, as the two databases were not linked using unique patient identifiers. Out of 996 diagnosed patients, 52.2% (n = 520) were found to have been placed on treatment, out of which 2 patients were excluded because their treatment start dates were not found. Of the 518 patients with treatment start dates, 51.2% (n = 265) were placed on treatment early; of the 253 placed on treatment late, 26.6% were between 31 and 60 days, 19.3% between 61–180 days and 2.9% >180 days. Fig 2 shows how study participants were selected and included.

Demographic statistics and outcomes for study participants are summarized in *Table 1*. The median age for the study population was 34.0, with males comprising 65.0%. Over half of the patients came from the South West and North Central regions, with 28.7% and 25.1% respectively, and were diagnosed or treated in an urban facility (52.8%). More patients were diagnosed in higher level facilities (State-owned = 39.9%; Reference lab = 31.7% and Federal Hospital = 21.4%) than in the primary care centers or private hospitals (1.6% and 5.4% respectively).

Some 2015 treatment enrolled patients (n = 140 and representing 21.2% of total treatment initiations) found in the treatment database could not be traced in the diagnosis (GxAlert) database and were excluded. Their characteristics are also included in Table 1. The median age was 37.0 and 77.1% were male. Most of the patients were from the South West (37.9%) and North West (25.7).

### Treatment initiation: Characteristics and predictors

At the national level, treatment initiation did not vary significantly between socio-demographic groups, although there were significant predictors at the geopolitical level. Among the 6 categorical variables tested, none had statistically significant associations with treatment initiation. However, logistic regression showed patients diagnosed in the South East (SE) and North East (NE) were less likely to be initiated on treatment compared to their counterparts in the South West (SW) (AOR: 0.57, 0.35–0.93 and 0.61, 0.37–1.00) respectively. Table 2 compares treatment initiation outcome for different socio-demographic groups and between different types of diagnostic facilities, and looks at predictors of early treatment initiation.

**Table 2:** National and regional characteristics and predictors of treatment initiation amongst 996 DR-TB patients diagnosed in 2015.

Individual and Community Level Characteristics		National level					SW Region only				
			Treatment initiated*		Predictors			Treatment initiated*		Predictors	
		N	Yes n(%)	No %	Crude OR (95% CI)	AOR** (95% CI)	N	Yes n(%)	No %	Crude OR (95% CI)	AOR** (95% CI)
Sex											
	Female	349	184 (52.7)	165 (47.3)	1.0 (reference)	-	105	59 (56.2)	46 (43.8)	1.0 (reference)	-
	Male	647	336 (51.9)	311 (48.1)	0.97 (0.73–1.26)		181	101 (55.8)	80 (44.1)	0.98 (0.61–1.60)	-
Age Groups											
	Age as a continuous variable	80	46 (57.5)	34 (42.5)	1.00 (0.99–1.02)	1.00 (0.99–1.02)				0.99 (0.98–1.01)	
	Children (0–19)	870	450 (51.7)	420 (48.3)	1.0 (reference)	-	24	17 (70.8)	7 (29.2)	1.0 (reference)	-
	Adults (20–59)	43	24 (55.8)	19 (44.2)	0.79 (0.50–1.26)	0.79 (0.50–1.26)	254	138 (54.3)	116 (45.7)	0.49 (0.20–1.22)	0.30 (0.10–0.96)
	Seniors (≥60)	80	46 (57.5)	34 (42.5)	0.93 (0.44–1.97)	0.93 (0.44–1.97)	8	5 (62.5)	3 (37.5)	0.69 (0.13–3.68)	0.32 (0.05–2.18)
Type of Diagnosis Facility											
	National/Zonal Laboratory	316	172 (54.4)	144 (45.6)	1.0 (reference)		115	68 (59.1)	47 (40.9)	1.0 (reference)	
	Federal Hospital	213	111 (52.1)	102 (47.9)	0.88 (0.65–1.18)		68	40 (58.8)	28 (41.2)	0.99 (0.54–1.82)	
	State-owned Hospital	397	203 (51.1)	194 (48.9)	0.84 (0.31–2.29)		100	51 (51.0)	49 (49.0)	0.72 (0.42–1.24)	
	Private Hospital	16	8 (50.0)	8 50.0)	0.78 (0.44–1.39)		3	1 (33.3)	2 (66.7)	0.35 (0.03–3.92)	
Urban/Rural†											
	Urban	526	274 (52.1)	252 (47.9)	1.0 (reference)	-					
	Semi-Urban	290	153 (52.8)	137 (47.2)	1.03 (0.77–1.37)	1.21 (0.89–1.66)					
	Rural	180	93 (51.7)	87 (48.3)	0.98 (0.70–1.38)	1.25 (0.86–1.81)					
Geopolitical zone											
	South West	286	160 (55.9)	126 (44.1)	1.0 (reference)						
	North Central	250	128 (51.2)	122 (48.8)	0.83 (0.59–1.16)	0.82 (0.58–1.17)					
	South South	143	75 (52.4)	68 (47.6)	0.87 (0.58–1.30)	0.88 (0.58–1.32)					
	North West	116	67 (57.8)	49 (42.2)	1.08 (0.70–1.67)	1.06 (0.68–1.65)					
	South East	107	48 (44.9)	59 (55.1)	0.64 (0.41–1.00)	0.57 (0.35–0.93)					
	North East	94	42 (44.7)	52 (55.3)	0.64 (0.40–1.02)	0.61 (0.37–1.00)					
States (SW region)											
	Ogun						36	27 (75.0)	9 (25.0)	1.0 (reference)	
	Ekiti						4	1 (25.0)	3 (75.0)	0.11 (0.01–1.20)	0.08 (0.01–0.93)
	Lagos						156	92 (59.0)	64 (41.0)	0.48 (0.21–1.09)	0.41 (0.18–0.96)
	Ondo						14	1 (7.1)	13 (92.9)	0.03 (0.003–0.22)	0.02 (0.002–0.15)
	Osun						19	10 (52.6)	9 (47.4)	0.31 (0.10–0.93)	0.26 (0.08–0.83)
	Oyo						57	29 (50.9)	28 (49.1)	0.35 (0.14–0.86)	0.30 (0.12–0.76)

* ‘Treatment initiated’ refers to whether patients diagnosed between January to December 2015 had been initiated on treatment as at August 2017

** Adjusted Odds Ratios (AOR) were obtained by comparing the likelihood of treatment initiation and the time-to-event using the Cox proportional hazard model, adjusted for age, sex, urban/rural or States, or geopolitical zones

† Some correlation was observed between the ‘Urb_Rur’ and ‘States’ variables during the test for collinearity, and the Urb_Rur was eliminated in order to build a stronger model

SW = South-West, n = number, OR = Odds Ratio, AOR = Adjusted Odds Ratio, CI = Confidence Interval

Further analysis in the SW zone showed similar locational differences, comparing treatment initiation differences between states in the region and for different socio-demographic groups. Among the 5 categorical variables tested, only the state within which patients were diagnosed showed statistical significance with treatment initiation. However, in adjusted logistic regression models, adults (ages 20–59) were less likely (AOR: 0.30, 0.10–0.96) than children (0–19) to be initiated on treatment. Also, Ogun State patients (as reference group) were also more likely to be initiated than the other states in this region. Patients in Ekiti and Ondo States were less likely (AOR: 0.08, 0.01–0.93 and 0.02, 0.002–0.15) to be initiated.

**Fig 3:**
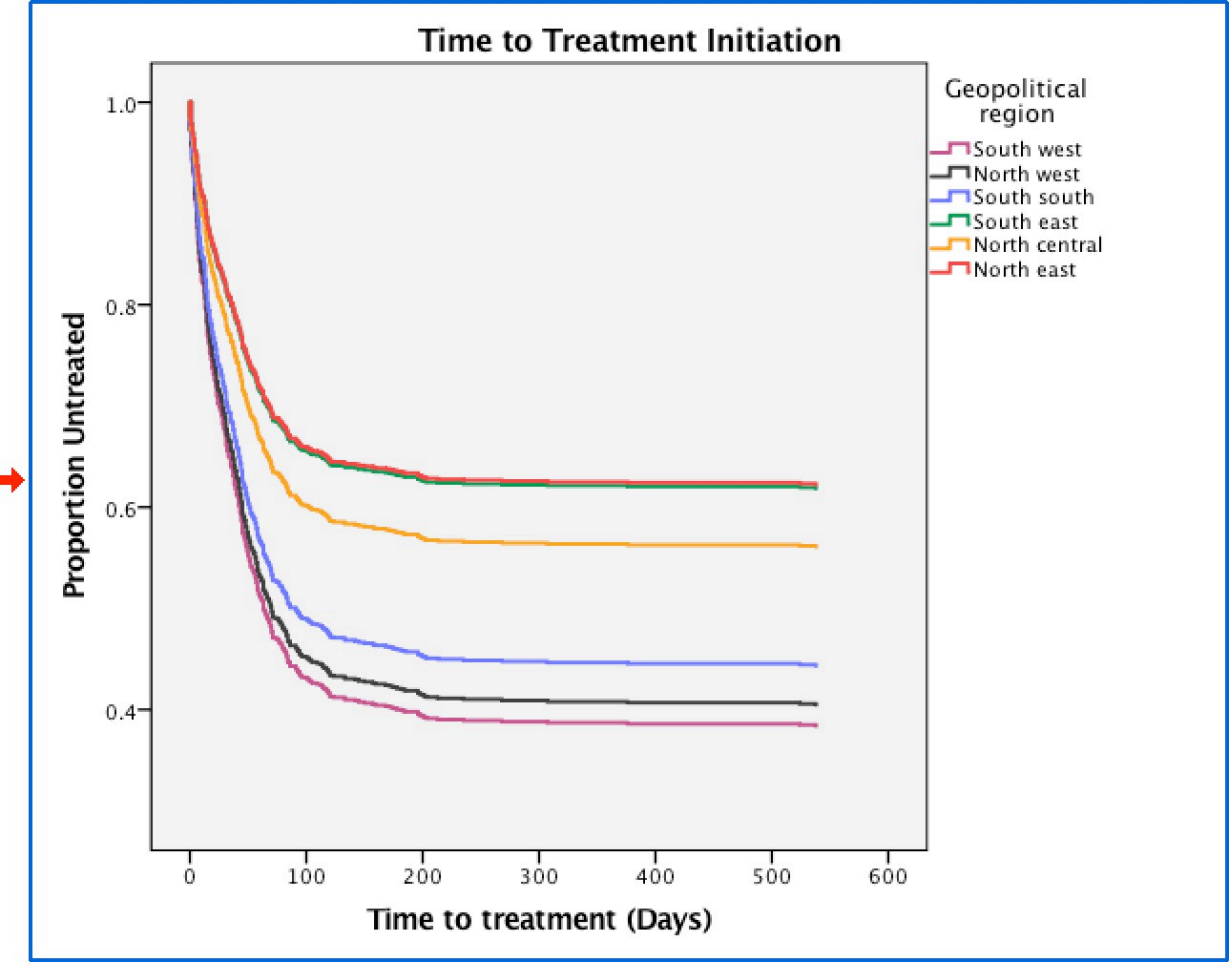
Time-to-treatment initiation in the Geopolitical zones, adjusted for sex, age and urban/rural.

Comparing the likelihood of treatment initiation and the time-to-event was done using the Cox proportional hazard model, and was adjusted for age, sex, urban/rural and geopolitical zones (Fig 3), as an examination of the log minus log plots did not show any of these covariates as violating the assumptions. In the Cox Proportional Hazard modeling time to treatment, patients in the NE were less likely to be treatment initiated (HR: 0.49, 0.34–0.72). Only the geopolitical zone was predictive of longer times to treatment in the adjusted model.

### Timely treatment: Characteristics and predictors

Geographical location (semi-urban, Ogun state), level of treatment initiation (facility-/community-based), and treatment history were significantly associated with timely treatment rates across different socio-demographic groups at the National level (Table 3).

**Table 3:** National and regional characteristics and predictors of treatment within 30 days amongst DR-TB diagnosed patients in 2015.

Individual and Community Level Characteristics		National level					SW Region only				
			Time to Treatment		Predictors			Time to Treatment		Predictors	
		N	< = 30 days n(%)	>30 days %	Crude OR (95% CI)	AOR * (95% CI)	N	< = 30 days n(%)	>30 days %	Crude OR (95% CI)	AOR* (95% CI)
Sex											
	Female	182	92 (50.5)	90 (49.5)	1.0 (reference)	-	73	34 (46.6)	39 (53.4)	1.0 (reference)	-
	Male	336	173 (51.5)	163 (48.5)	1.04 (0.73–1.49)	-	121	68 (56.2)	53 (43.8)	1.47 (0.82–2.64)	1.86 (0.93–3.40)
Age Groups											
	Age as a continuous variable	46	20 (43.5)	26 (56.5)	0.99 (0.98–1.01)					1.00 (0.98–1.03)	
	Children (0–19)	448	235 (52.5)	213 (47.5)	1.0 (reference)	-	19	9 (47.4)	10 (52.6)	1.0 (reference)	
	Adults (20–59)	24	10 (41.7)	14 (58.3)	1.43 (0.78–2.64)	-	168	88 (52.4)	80 (47.6)	1.22 (0.47–3.16)	
	Seniors (≥60)	46	20 (43.5)	26 (56.5)	0.93 (0.34–2.52)	-	7	5 (71.4)	2 (28.6)	2.78 (0.43–18.04)	
Treatment level											
	Community-based	261	112 (42.9)	149 (57.1)	1.0 (reference)	-	83	27 (32.5)	56 (67.5)	1.0 (reference)	-
	Facility-based	257	153 (59.5)	104 (40.5)	1.96 (1.38–2.78)	1.77 (1.20–2.61)	111	75 (67.6)	36 (32.4)	4.32 (2.35–7.93)	0.48 (0.14–1.71)
Treatment History											
	New	97	58 (59.8)	39 (40.2)	1.0 (reference)	-	18	13 (72.2)	5 (27.8)	1.0 (reference)	-
	Retreatment	224	106 (47.3)	118 (52.7)	0.60 (0.37–0.98)	0.59 (0.35–0.99)	104	58 (55.8)	46 (44.2)	0.49 (0.16–1.46)	0.27 (0.07–1.00)
	Relapsed	114	65 (57.0)	49 (43.0)	0.89 (0.52–1.55)	0.92 (0.51–1.65)	32	22 (68.8)	10 (31.3)	0.85 (0.24–3.02)	0.60 (0.14–2.56)
	Default	57	20 (35.1)	37 (64.9)	0.36 (0.18–0.72)	0.47 (0.23–0.99)	34	5 (14.7)	29 (85.3)	0.07 (0.02–0.27)	0.08 (0.02–0.41)
	Unknown History	26	16 (61.5)	10 (64.9)	1.08 (0.44–2.62)	0.95 (0.37–2.43)	6	4 (66.7)	2 (33.3)	0.77 (0.11–5.61)	0.61 (0.06–6.10)
Type of Resistance											
	Mono Resistant	81	38 (46.9)	43 (53.1)	1.0 (reference)	-	21	10 (47.6)	11 (52.4)	1.0 (reference)	
	Rif resistant	107	56 (52.3)	51 (47.7)	1.24 (0.70–2.22)		45	22 (48.9)	23 (51.1)	1.05 (0.37–2.97)	
	Multidrug resistant	268	140 (52.2)	128 (47.8)	1.24 (0.75–2.04)		100	54 (54.0)	46 (46.0)	1.29 (0.50–3.31)	
	Poly resistant	62	31 (50.0)	31 (50.0)	1.13 (0.58–2.19)		28	16 (57.1)	12 (42.9)	1.47 (0.47–4.57)	
Urban/Rural**											
	Urban	308	138 (44.8)	170 (55.2)	1.0 (reference)	-					
	Semi-Urban	135	88 (65.2)	47 (34.8)	2.31 (1.52–3.51)	2.11 (1.34–3.32)					
	Rural	75	39 (52.0)	36 (48.0)	1.34 (0.81–2.21)	1.63 (0.87–3.05)					
Geopolitical zone											
	South West	194	102 (52.6)	92 (47.4)	1.0 (reference)	-					
	North Central	98	47 (48.0)	51 (52.0)	0.83 (0.51–1.35)	0.72 (0.42–1.23)					
	South South	87	42 (48.3)	45 (51.7)	0.84 (0.51–1.40)	0.68 (0.40–1.17)					
	North West	70	42 (60.0)	28 (40.0)	1.35 (0.78–2.36)	1.27 (0.70–2.31)					
	South East	38	18 (47.4)	20 (52.6)	0.81 (0.41–1.63)	0.73 (0.31–1.70)					
	North East	31	14 (45.2)	17 (54.8)	0.74 (0.34–1.59)	0.58 (0.25–1.32)					
States†											
	Ogun						53	40 (75.5)	13 (24.5)	1.0 (reference)	-
	Lagos						82	23 (28.0)	59 (72.0)	0.13 (0.06–0.28)	0.10 (0.03–0.41)
	Osun						10	4 (40.0)	6 (60.0)	0.22 (0.05–0.89)	0.18 (0.03–0.95)
	Oyo						49	35 (71.4)	14 (28.6)	0.81 (0.34–1.96)	1.12 (0.44–2.83)

* Adjusted Odds Ratios (AOR) were obtained by comparing the likelihood of treatment initiation and the time-to-event using the Cox proportional hazard model, adjusted for age, sex, urban/rural or States, or geopolitical zones

**Some correlation was observed between the ‘Urb_Rur’ and ‘States’ variables during the test for collinearity, and the Urb_Rur was eliminated in order to build a stronger model

SW = South-West, n = number, OR = Odds Ratio, AOR = Adjusted Odds Ratio, CI = Confidence Interval, Rif = Rifampicin

† Ekiti and Ondo States had less than 3 patients each and were excluded from the analysis

Patients living in semi-urban areas and patients initiating as in-patients in a hospital were more likely to experience timely initiation of treatment than those in urban areas or as outpatients (AOR: 2.11, 1.34–3.32, 1.77, 1.20–2.61).

In the SW zone (Table 3), State of residence, treatment initiation level (facility or community), and treatment history were significantly associated with timely treatment, much like the national level data. Ogun state residence, treatment initiation in a treatment center, and treatment-naivety were predictive of earlier treatment initiation.

## Discussion

The WHO recommends early treatment initiation within 4 weeks of DR-TB diagnosis[5] and this is prescribed the Nigerian DR-TB treatment guidelines. [23] Timely diagnosis and treatment initiation is critical for the control of DR-TB in Nigeria, one of the major contributors to the global burden of DR-TB. Our review of current literature did not find any study on the timeliness of or time to DR-TB treatment in Nigeria.

This study investigated predictors for treatment initiation and timely treatment among DR-TB patients diagnosed in 2015 in Nigeria. On average, only 3.46% of estimated DR-TB patients in Nigeria were diagnosed, only half of these were treated at all and only 1 in 4 of these received treatment within the 30 days after diagnosis as recommended by the Nigerian NTP and WHO. Our findings highlight the need to improve the timeliness of DR-TB treatment for patients in Nigeria in order that efforts to improve case detection not result in large numbers of diagnosed but untreated patients.

Geographical location of the DR-TB facility emerged as a major factor in DR-TB treatment initiation and timeliness in our study, similar to other studies in sub-Saharan Africa (SSA). [24–26] Patients in some regions were half as likely to be ever initiated on treatment as those in the SW region. Timeliness of treatment did not vary significantly except for two States (Lagos and Osun) where patients were up to 10 times less likely to be treated within 1 month.

These regional differences were likely due to program immaturity and insecurity in some regions compared to others. With the Global Fund (GFATM) supported scale-up of DR-TB services in 2011, the SW saw the first set of reference laboratories, treatment centers with large bed capacities and community treatment enrolment, with patient referrals from other parts of Nigeria. However, another region, the NE, was slow to activate services due to increasing security challenges and frequent insurgent activities between 2013–2015, severely hampering healthcare. In the SW, the treatment centre in Lagos State was shut down as a result of the Ebola crisis in 2015 while the treatment centre in Ogun State was not activated until the last quarter of 2015.

Community treatment initiation was rapidly scaled up in the 2nd quarter of 2015 to mitigate challenges patients were facing when admitted to treatment centers away from family for 8 months. Patients had to meet certain criteria such as clinical stability to be eligible for community initiation. The in-patient treatment centers were reserved mostly for children, pregnant women, critically ill patients and those with co-morbidities. This may have contributed to patients in the facility being more likely to initiate timely treatment than those in the community, although community-based management has the potential to reduce treatment delays in similar settings.[27]

Since the study period, Nigeria, in collaboration with GFATM and partners, have scaled-up DRTB services to include 29 treatment sites (up from 16 in 2015), 37 GeneXpert sites reference labs (up from 34) and community PMDT in all 37 states (up from 27 states in 2015).[14] As a result of this, it is likely that average times to treatment initiation are now shorter but new national treatment initiation data have not yet been released. In addition, contextual and multilevel analyses of regional and sub-regional inequalities in access to timely care are needed to inform policy and practice.

The WHO TB Report 2018[6] shows that rates of diagnosis and treatment in Nigeria remain low. If the supply end—diagnostic and treatment services—are improving, a corresponding improvement in rates and timeliness of treatment initiation is expected. Where these are not observed, more attention needs to be paid to the demand end- patient sociodemographic characteristics that influence access to care. Further research is needed on the trends in time to treatment in Nigeria, and the patient factors that underlie these.

### Study limitations

Our study had several limitations. Firstly, the excluded 140 patients on treatment could have contributed more to our understanding of timely treatment predictors. Secondly, the two source documents (diagnosis and treatment databases) were not linked with unique patient identifiers and had to be matched manually by other data points. This could have led to some errors in the numbers reported in each category. Finally, due to the retrospective nature of this study, we could not account for the effect of other patient socio-demographic characteristics such as income level, religion and culture, marital status on the risk of being untreated or delayed treatment as this information were not recorded in our source databases.

Preliminary discussions with programme managers, including coauthors of this manuscript, have suggested a range of possible contextual reasons for these state-level differences–including the conversion of the Lagos state DR-TB treatment centre into an Ebola hospital in 2014—but further research is needed to understand these contextual factors.

## Conclusions

Timely access to diagnosis and treatment services are critical to DR-TB patients’ survival and other treatment outcomes. However, within Nigeria, nearly half of diagnosed DR-TB patients are never initiated on treatment, less than a quarter initiate treatment within the recommended 30 days after diagnosis, and regional inequalities in access to DR-TB care are significant. In a context where DR-TB case detection is less than 5% of the estimated burden, these low rates of treatment for cases already detected pose both practical and ethical challenges to DR-TB control. Disparities persist in access to services at patient, facility, and geopolitical levels. Semi-urban and rural residence as well as being initiated in the facility were predictive of earlier treatment initiation, suggestive of complex access challenges. The geographic location of the diagnosing or treatment facility were significantly associated with treatment initiation and timely treatment highlighting significant geopolitical and state differences. The National TB Program needs to strengthen linkages between diagnosis and treatment, as well as provide adequate access and timely treatment initiation for DR-TB patients, especially in the communities, across all geopolitical zones and states.

## Supporting information

S1 Table Variables in the diagnosis database (GX Alert). (DOCX)

S2 Table Variables in the treatment database (E-TB Manager). (DOCX)

S1 Fig Estimated TB/DR-TB National case notification rates by geopolitical zones in Nigeria*. *Study DR-TB data on number of diagnosed patients per state were used to calculate DR-TB case notification rates based on 2015 estimates. These were compared with the National TB case notification rates for the same year.
(TIF)

S2 Fig Estimated TB and DR-TB CDR by South-Western States of Nigeria*. *Comparing number of diagnosed patients and case notification rates also showed significant regional differences.
(TIF)
