# Supplementary material for: Rates and timeliness of treatment initiation among drug-resistant tuberculosis patients in Nigeria- A retrospective cohort study
Source: PLoS One. 2019 Apr 25;14(4):e0215542. doi: 10.1371/journal.pone.0215542 (PMC6483179; doi:10.1371/journal.pone.0215542)
Supplement: S1 Table — (DOCX) [file pone.0215542.s001.docx]

**S1 Table: Variables in the Diagnosis Database (GX Alert)**

| **Patient ID** | **Test Notes** | | **** Age groups** | **Test Facility** | ****Type of Diagnosis Facility** | **Test State** | ****Geopolitical zone** | ****Urban/Rural** | **Test Date** | ****Test Quarter** | *****Treatment initiated** |
| --- | --- | --- | --- | --- | --- | --- | --- | --- | --- | --- | --- |
|  | ***Age** | ***Sex** |  |  |  |  |  |  |  |  |  |
|  |  |  |  |  |  |  |  |  |  |  |  |
|  |  |  |  |  |  |  |  |  |  |  |  |
|  |  |  |  |  |  |  |  |  |  |  |  |
|  |  |  |  |  |  |  |  |  |  |  |  |
|  |  |  |  |  |  |  |  |  |  |  |  |
|  |  |  |  |  |  |  |  |  |  |  |  |
|  |  |  |  |  |  |  |  |  |  |  |  |
|  | | | | | | | | | | | |

* The variables – age and sex- were created from the information recorded in the test notes

** Recoded variables

*** Derived after data linkage to treatment database

For additional information about this database, go to [www.ntblcp.org.ng/](http://www.ntblcp.org.ng/)
