# Supplementary material for: Rates and timeliness of treatment initiation among drug-resistant tuberculosis patients in Nigeria- A retrospective cohort study
Source: PLoS One. 2019 Apr 25;14(4):e0215542. doi: 10.1371/journal.pone.0215542 (PMC6483179; doi:10.1371/journal.pone.0215542)
Supplement: S2 Table — (DOCX) [file pone.0215542.s002.docx]

**S2 Table: Variables in the Treatment Database (E-TB Manager)**

| **Patient ID** | **Sex** | **Age** | *** Age groups** | **Registration unit** | ***Treatment level** | **Geopolitical zone** | **Registration State** | **Urban/ Rural** | **Date of registration** | **Diagnosis date** | **Treatment Start date** | **Type of resistance** | **Treatment history** |
| --- | --- | --- | --- | --- | --- | --- | --- | --- | --- | --- | --- | --- | --- |
|  |  |  |  |  |  |  |  |  |  |  |  |  |  |
|  |  |  |  |  |  |  |  |  |  |  |  |  |  |
|  |  |  |  |  |  |  |  |  |  |  |  |  |  |
|  |  |  |  |  |  |  |  |  |  |  |  |  |  |
|  |  |  |  |  |  |  |  |  |  |  |  |  |  |
|  | | | | | | | | | | | | | |

* Recoded variables

** After data linkage with the diagnosis database

Excluded variables- Patient name, patient address, First medical examination date, Treatment end date, initial weight, case definition, type of disease, number of previous TB treatment, site of disease, registration local government (LGA)

For additional information about this database, go to [www.ntblcp.org.ng/](http://www.ntblcp.org.ng/)
