# Supplementary figures and images for: Rates and timeliness of treatment initiation among drug-resistant tuberculosis patients in Nigeria- A retrospective cohort study
Source: PLoS One. 2019 Apr 25;14(4):e0215542. doi: 10.1371/journal.pone.0215542 (PMC6483179; doi:10.1371/journal.pone.0215542)

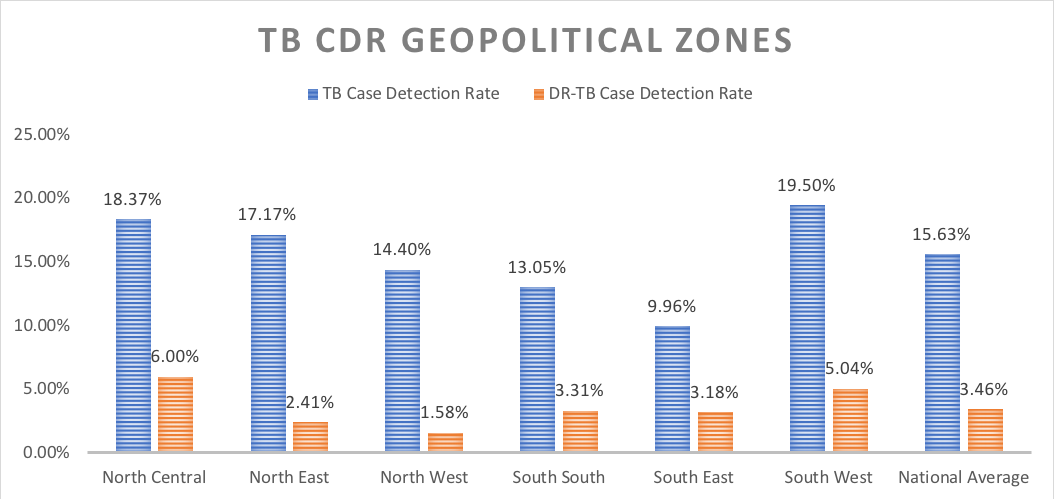

Supplement: S1 Fig — *Study DR-TB data on number of diagnosed patients per state were used to calculate DR-TB case notification rates based on 2015 estimates. These were compared with the National TB case notification rates for the same year. (TIF) [file pone.0215542.s003.tif]

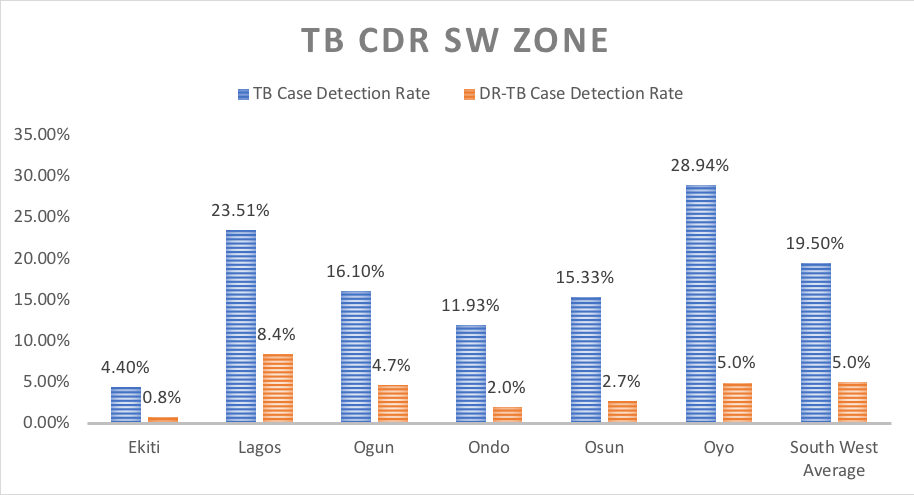

Supplement: S2 Fig — *Comparing number of diagnosed patients and case notification rates also showed significant regional differences. (TIF) [file pone.0215542.s004.tif]
